# Rheological Property Modification of a Molten-State Polyamide through the Addition of an *α*-Olefin–Maleic Anhydride Copolymer

**DOI:** 10.3390/molecules29163730

**Published:** 2024-08-06

**Authors:** Xianzhu Mei, Quoc-Viet Do, Takaaki Narita, Misaki Yamaguchi, Masayuki Yamaguchi

**Affiliations:** 1 Materials Chemistry Frontiers Research Area, Japan Advanced Institute of Science and Technology, 1-1 Asahidai, Nomi 923-1292, Ishikawa, Japan; s2210026@jaist.ac.jp (X.M.); doquocviet@jaist.ac.jp (Q.-V.D.); 2 Performance Additives Group, C&A Hiroshima, Coating & Additives R&D Center, Mitsubishi Chemical Corporation, 20-1 Miyukicho, Otake 739-0693, Hiroshima, Japan; takaaki.narita.ma@mcgc.com (T.N.); misaki.yamaguchi.ma@mcgc.com (M.Y.)

**Keywords:** rheology modification, polymer processing, elongational viscosity, melt elasticity, polyamide

## Abstract

The rheological properties of a polyamide (PA) resin with low crystallinity were modified by melt-mixing it with a small amount of an alternative *α*-olefin–maleic anhydride copolymer as a reactive compound. Because PA has a low melting point, rheological characterization was performed over a wide temperature range. Owing to the reaction between PA and the alternative *α*-olefin–maleic anhydride copolymer, the blend sample behaved as a long-chain branched polymer in the molten state. The thermo-rheological complexity was obvious owing to large flow activation energy values in the low modulus region, i.e., the rheological time–temperature superposition principle was not applicable. The primary normal stress difference under steady shear was greatly increased in the wide shear rate range, leading to a large swell ratio at the capillary extrusion. Furthermore, strain hardening in the transient elongational viscosity, which is responsible for favorable processability, was clear. Because this is a simple modification method, it will be widely employed to modify the rheological properties of various polyamide resins.

## 1. Introduction

It is essential that engineering plastics including polyamides are able to undergo various processing operations besides injection molding. Most engineering plastics have a narrow molecular weight distribution and no long-chain branches. Therefore, during processes in which a molten resin with a free surface is deformed, it is necessary to modify the rheological properties of the resin to provide strain hardening in transient elongational viscosity. Such processes include foaming, blow molding, thermoforming, T-die casting, and tubular-blown film processing. Various techniques have been proposed to achieve this modification: (1) the addition of an ultra-high molecular-weight fraction [1,2,3], (2) the incorporation of long-chain branches [4,5,6], (3) the addition of flexible nanofibers [7,8,9], (4) the addition of a critical gel [10,11], (5) the addition of a comb-shaped block copolymer in an immiscible polymer blend [12,13], (6) blending with an immiscible long-chain branched polymer [14,15], and (7) making a multi-layered structure [16].

**Figure 1:**
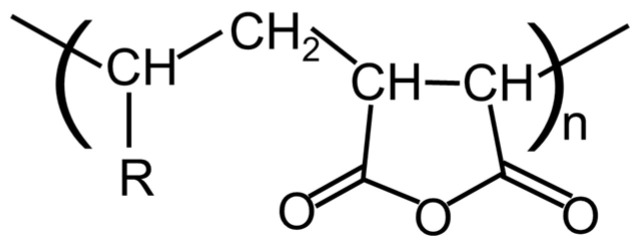
Chemical structure of the alternative α-olefin–maleic anhydride copolymer (O-MAH).

Polyamides are engineering plastics with high mechanical toughness, abrasion resistance, heat resistance, oil resistance, and gas barrier properties [17,18,19]. They are therefore potentially very useful for various products such as films, bottles, and foams. Reaction at the amide groups of polyamides is a good method of providing long-chain branches and therefore strain hardening in elongational viscosity [20]. Among various chain extenders [21,22,23,24,25,26,27,28,29], maleic anhydride is one of the most appropriate reactive compounds. Maleic anhydride graft copolymers such as polypropylene–graft–maleic anhydride (PP-g-MAH) are widely available and are used as compatibilizers for blends comprising polypropylene (PP) and polyamides [30,31,32]. This is logical because the polymer chains of the graft copolymer are localized at the boundary between the two phases and reduce the possibility of the coalescence of dispersed droplets [33,34]. However, such a graft copolymer is not appropriate for the rheological modification of a polyamide resin because one component of the graft copolymer, e.g., PP in PP-g-MAH, is immiscible with the polyamide resin. Therefore, PP exists as a dispersed phase. An alternative copolymer of *α*-olefin and maleic anhydride (O-MAH, shown in Figure 1), which reacts readily with polyamides, is a good candidate of a rheology modifier because the *α*-olefin component in the alternative copolymer must be impossible to show phase separation. In fact, Li et al. employed O-MAH to modify polyamide 6 (PA6) and confirmed that its foaming processability was improved [29].

However, it is not easy to characterize the rheological properties of conventional polyamides such as PA6 and polyamide 66 (PA66), especially in the low frequency region, owing to severe thermal degradation beyond their melting points [35,36,37,38]. Elongational viscosity is also difficult to evaluate [20,39,40]. Therefore, in the present study, a copolyamide resin with low crystallinity, denoted as PA, was used for the evaluation of basic rheological properties at relatively low temperatures. Subsequently, the effect of the addition of O-MAH on the rheological properties of the polyamide was discussed in detail. In particular, thermo-rheological simplicity/complexity and transient elongational viscosity were evaluated in detail because they are known to be sensitive to long-chain branch structures [4,5,6,41]. The results obtained herein will be widely available to inform modification of the rheological properties and processabilities of various polyamide resins.

## 2. Results and Discussion

**Figure 2:**
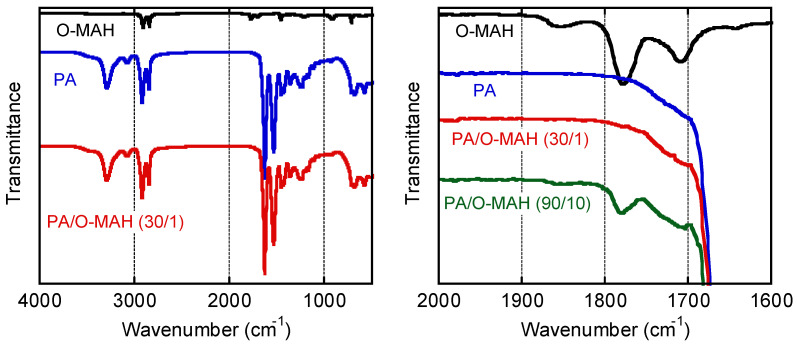
ATRIR spectra of the samples.

Figure 2 shows the IR spectra of O-MAH, PA, and their blends. Stretching vibrations attributable to the carbonyl groups of O-MAH were detected at 1780 and 1850 cm^−1^ [29]. These peaks disappeared in PA/O-MAH (30/1), demonstrating that all the maleic anhydride in O-MAH had reacted with the amide groups in the PA during mixing and compression molding. In the previous study [29], it was confirmed that the bands of amide groups became weak after melt-mixing. In this study, however, it was not obvious because of the strong intensity. Although exposure to high temperature results in the dehydration of amide bonds in polyamide resins [42], it hardly occurred in this experiment because of the low temperature mixing at 140 °C. In fact, the PA sample in Figure 2 had the same thermal history. The stretching vibrations in PA/O-MAH (90/10) weakened but did not disappear. Although some of the maleic anhydride reacted with the PA, some remained in the blend. It suggested that 10 wt% of O-MAH was too much for the present PA sample. Subsequently, therefore, we employed only PA/O-MAH (30/1) as the blend sample because further reactions barely occurred during the rheology measurements.

**Figure 3:**
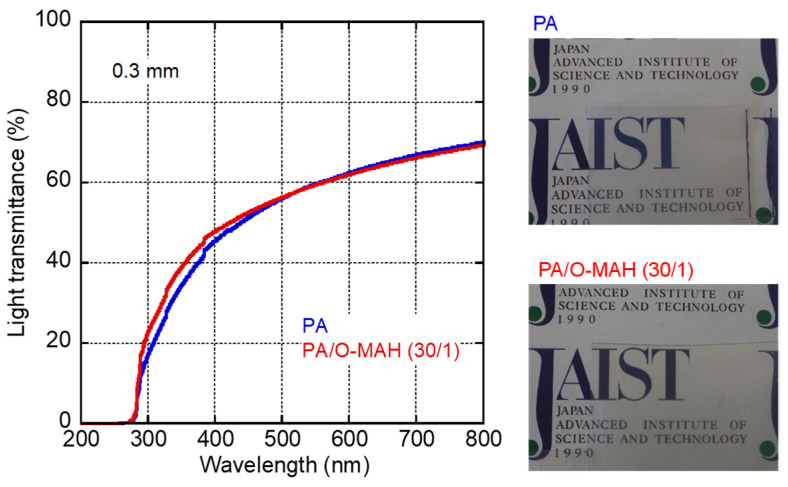
Light transmittance as a function of the wavelength of PA and PA/O-MAH (30/1) films (0.3 mm thick). Photographs of the films are shown on the right.

The light transmittance of each 0.3 mm-thick PA and PA/O-MAH (30/1) film was evaluated using a UV-vis spectrometer, and the results are shown in Figure 3. Photographs of the films are also shown in the figure. The light transmittance increased with wavelength, indicating light absorption in the short wavelength range [35]. Considering that approximately 10% of the light transmittance was lost by surface reflection [43,44], both films were relatively transparent, as demonstrated by the photographs. This must have been due to the lower light scattering of the crystalline structure of PA [45,46,47]. Moreover, the O-MAH addition hardly affected the transparency. The result suggested that the extent of phase separation, if any, must have been small in the blend, and we could not detect any phase-separated structure in the blend by electron microscopy.

**Figure 4:**
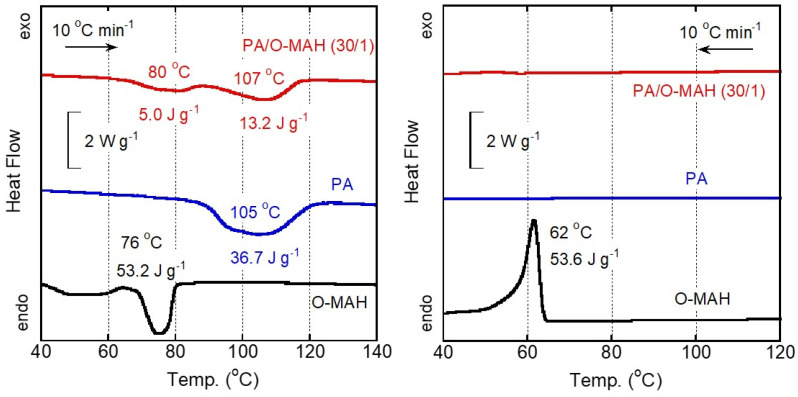
DSC heating (**left**) and cooling (**right**) curves at 10 °C min^−1^ produced by PA, O-MAH, and PA/O-MAH (30/1).

The DSC heating and cooling curves are shown in Figure 4. The heating/cooling rate was 10 °C min^−1^. There was an endothermic melting peak at approximately 105 °C in the DSC curve of pure PA, and the heat of fusion of 36.7 J g^−1^. Assuming that these crystals were composed of the main component in PA, i.e., polyamide 6 (PA6), the crystallinity was calculated to be 19% (the heat of fusion of perfect PA6 crystals was reported to be 230 J g^−1^) [48]. Because PA crystallization occurred slowly, no crystallization peak was detected at this cooling rate. In the case of pure O-MAH, a main melting peak was detected at approximately 76 °C, with a weak broad/shoulder peak at approximately 55 °C. These melting peaks can be attributed to the crystallinity of the α-olefin, as reported previously [49,50,51,52]. There was a clear crystallization peak at 62 °C in the cooling curve. The PA/O-MAH (30/1) film produced two peaks in the heating curve at approximately 80 °C and 107 °C. The PA crystallinity was reduced by adding O-MAH. Moreover, a crystallization peak was absent from the curve generated by the blend film.

**Figure 5:**
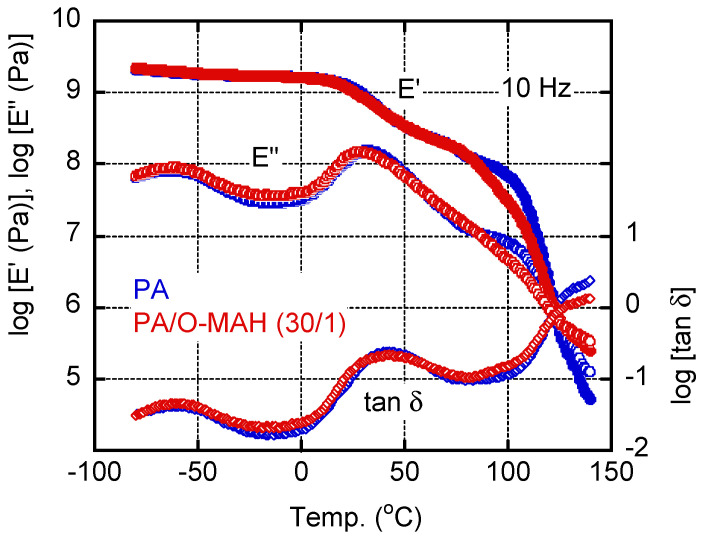
Temperature dependencies of the tensile storage modulus *E*′, loss modulus *E*″, and loss tangent *tan δ* at 10 Hz of the PA and PA/O-MAH (30/1) films.

Figure 5 shows the temperature dependencies of the tensile storage modulus *E*′, loss modulus *E*″, and loss tangent *tan δ* of the PA and PA/O-MAH (30/1) films. There was a peak in the *E*″ curve at approximately −60 °C (−100 °C to −30 °C), which can be attributed to localized segmental motion [53,54]. This relaxation mode was not affected by the addition of O-MAH. The *E*′ values decreased from approximately 20 °C owing to the glass-to-rubber transition of PA. Correspondingly, there was a peak in the *E*″ curve as well as the *tan δ* curve. The peak temperature of *E*″, i.e., the glass transition temperature *T_g_*, of the PA film was approximately 30 °C, which was lower than that of PA6 [35]. The *T_g_* of the PA/O-MAH (30/1) film was slightly lower. The decrease in the *T_g_* following the addition of O-MAH was presumably attributable to its lower crystallinity, which increased segmental motion. The *E*′ decreased gradually, indicating that the PA was not fully amorphous, as shown in Figure 4. Beyond 100 °C, *E*′ dropped abruptly owing to the melting of the PA crystals. The *E*′ decrease occurred at 85 °C in the blend film. This must be attributed to the melting of the O-MAH crystals. 

**Figure 6:**
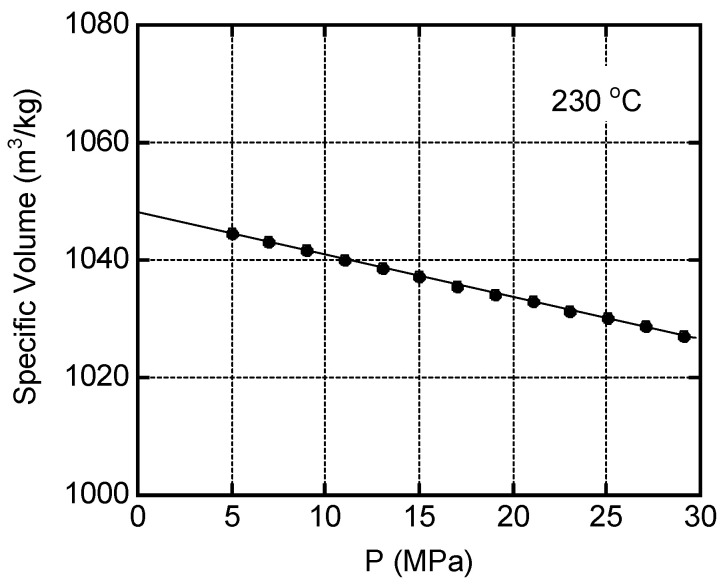
Specific volume of PA as a function of pressure, *P*, at 230 °C.

To understand the basic characteristics of PA in the molten state, we evaluated the relationship between pressure and specific volume at 230 °C. As shown in Figure 6, a straight line was obtained. The slope provided the bulk modulus *K*, which was 1.47 GPa and was determined by the following relationship [55]:

(1) $$K=\frac{P}{\left(\Delta V/V_{0}\right)},$$

where *V*_0_ is the volume at *P* = 0.

The melt density *ρ* at atmospheric pressure was calculated to be 954 kg m^−3^.

**Figure 7:**
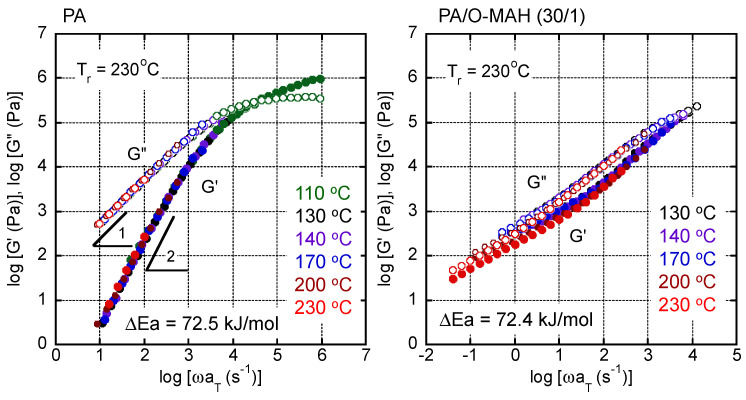
Master curves of oscillatory shear storage modulus *G*′ and loss modulus *G*″ of PA (**left**) and PA/O-MAH (30/1) (**right**) at the reference temperature, *T*_r_, of 230 °C.

The master curves of the angular frequency dependency of the oscillatory shear moduli, such as shear storage modulus *G*′ and loss modulus *G*″, of PA and PA/O-MAH (30/1) are shown in Figure 7. The reference temperature was 230 °C. Although the time–temperature superposition principle was not applicable to PA/O-MAH (30/1), as explained in detail later, the apparent flow activation energy Δ*E*_a_ was roughly calculated according to the Arrhenius equation, and was determined to be 72.4 kJ mol^−1^ for PA/O-MAH (30/1). This value was almost the same as that of pure PA, i.e., 72.5 kJ mol^−1^. The measurement was also performed at 130 °C in the present experiment. There were no crystals at that temperature because the time–temperature superposition principle was applicable to PA.

As shown in Figure 7, the rheological terminal region was clearly detected in PA. The slopes of *G*′ and *G*″ in the low-frequency region were 2 and 1, respectively. The zero-shear viscosity *η*_0_ and the steady-state shear compliance *J_e_*^0^ were calculated using the following equations [55]:

(2) $$\eta_{0}=\underset{\omega \to 0}{\lim}\frac{G^{\prime \prime }}{\omega },$$

(3) $$J_{e}^{0}=\underset{\omega \to 0}{\lim}\frac{G^{\prime }}{G^{\prime \prime }},$$

The rheological terminal parameters at 230 °C were as follows: *η*_0_ = 53.4 Pa s and *J_e_*^0^ = 1.1 × 10^−5^ Pa^−1^. For PA/O-MAH, neither parameter was obtained in the measurement range, suggesting a long-time relaxation mechanism.

As is well known, the rubbery plateau modulus *G_N_*^0^ of a simple polymer melt can be calculated using the following equation [55]:

(4) $$G_{N}^{0}=\frac{2}{\pi }\int_{-\infty }^{a}G^{\prime \prime }d\ln\omega ,$$

where *a* is the angular frequency at the upper limit of the terminal region. The value of $G_{N}^{0}$ is usually obtained by doubling the numerical integration of *G*″ from ln *⍵* = −∞ to the maximum of *G*″. The $G_{N}^{0}$ of PA is 2.1 MPa. Because the melt density, *ρ*, was known, the average molecular weight between entanglement coupling by points, i.e., *M*_e_, can be calculated from $G_{N}^{0}$ [55]:

(5) $$M_{e}=\frac{\rho RT}{G_{N}^{0}},$$

where *R* is the gas constant.

The *M*_e_ value of the PA used in the present study was 1900. This value is close to those of other aliphatic polyamide resins, such as PA6 (*M*_e_ = 2490) and PA66 (*M*_e_ = 2000) [56].

**Figure 8:**
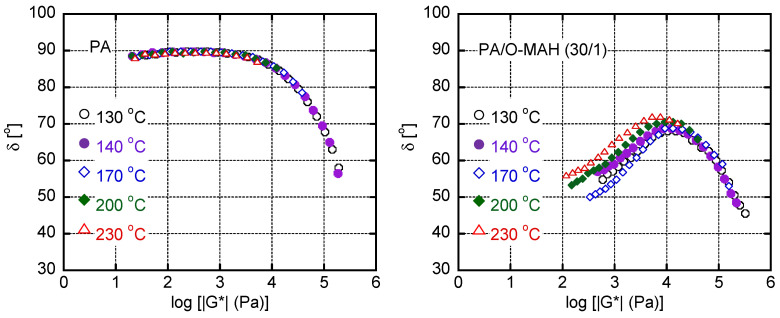
van Gurp–Palmen plots of PA and PA/O-MAH (30/1). The data were obtained at 130, 140, 170, 200, and 230 °C.

Considering the thermo-rheological complexity detected in PA/O-MAH (30/1), the van Gurp–Palmen plot, i.e., loss angle *δ* versus absolute value of complex shear modulus |G*|, is shown in Figure 8. A curve that was typical of most simple polymer liquids was obtained for PA without deviation at any temperature. In contrast, PA/O-MAH (30/1) exhibited a significantly different trend. Although it was possible to superimpose the data obtained at various temperatures onto each other in the high |G*| region, the deviation was obvious in the low |G*| region. The *δ* value decreased as the measurement temperature decreased. This phenomenon has often been detected in long-chain branched polymers owing to the large activation energy in the low modulus region [57,58]. Therefore, the present results demonstrated that the reactions between PA and O-MAH generated long-chain branches.

**Figure 9:**
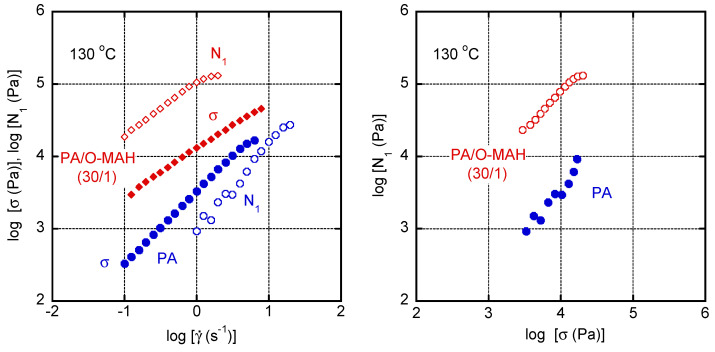
(**Left**) Shear stress *σ* and primary normal stress difference *N*_1_ as a function of shear rate $\dot{\gamma }$; and (**right**) *N*_1_ versus *σ* for PA and PA/O-MAH (30/1) at 130 °C.

Figure 9 shows the shear rate $\dot{\gamma }$ dependence of shear stress *σ* and primary normal stress difference *N*_1_ at 130 °C. Both *σ* and *N*_1_ increased with increasing $\dot{\gamma }$ for the samples. In addition, the values of *N*_1_ for PA/O-MAH (30/1) were much higher than those for pure PA over the whole shear-rate region. Furthermore, it should be noted that the order of *σ* and *N*_1_ was reversed, i.e., *σ* > *N*_1_ for PA and *σ* < *N*_1_ for the blend, demonstrating that the addition of O-MAH greatly enhanced elasticity over viscosity. This behavior was clearly confirmed by the right figure.

**Figure 10:**
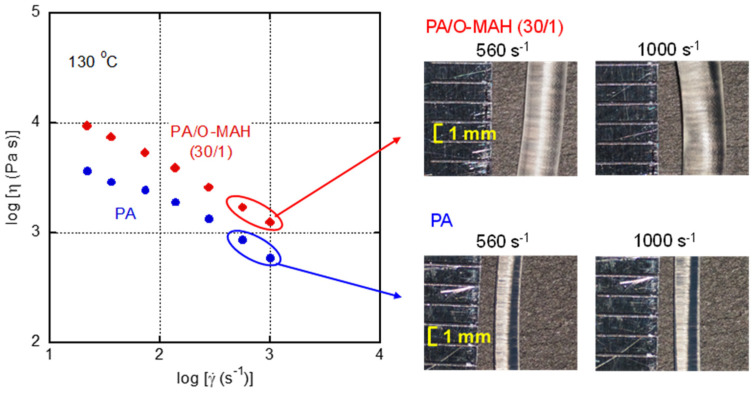
Steady-state shear viscosity *η* of PA and PA/O-MAH (30/1) at 130 °C. The photographs show strands extruded at 560 and 1000 s^−1^ through a circular die with a diameter of 1 mm.

In the high shear rate region, a capillary rheometer was employed to evaluate the steady-state shear viscosity *η* and the appearance of the extruded strands. In the present study, neither Bagley nor Rabinowitsch corrections were performed. As shown in Figure 10, the steady-state shear viscosity was enhanced to some degree by the addition of O-MAH. However, flow instabilities such as shark-skin failure and volumetric gross melt fracture were absent over the whole shear rate range. This result suggested that the applied shear stress at die exit and elongational stress at die entry were still lower than their critical onset values of flow instabilities [6,59,60,61].

As revealed by the photographs, the diameters of the extruded strands of PA/O-MAH (30/1) were much larger than those of PA, demonstrating that the melt elasticity was greatly increased by the addition of O-MAH. These results corresponded with those in Figure 9.

**Figure 11:**
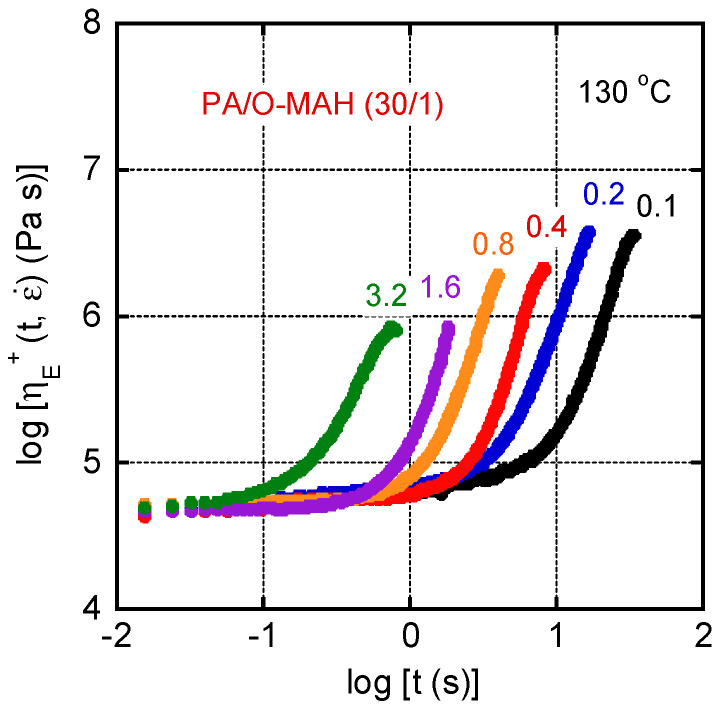
Growth curves of the transient elongational viscosity *η_E_*^+^ of PA/O-MAH (30/1) at 130 °C. The numerals in the figure represent the strain rates, $\dot{\varepsilon }$.

Figure 11 shows the growth curves of the uniaxial elongational viscosity *η_E_*^+^ at 130 °C of PA/O-MAH (30/1). It was impossible to evaluate the *η_E_*^+^ of pure PA owing to its severe downward deformation due to gravitational force. The numerals in the figure represent the elongational strain rates, $\dot{\varepsilon }$. Since the vertical and horizontal axes are proportional to stress and strain, respectively, they are basically stress–strain curves in double-logarithm plots [14]. There was marked strain hardening, i.e., sudden increase in *η_E_*^+^, even at a low strain rate. This is typical for a polymer melt with a well-developed long-chain branch structure [4,5,6,30,59,60]. Chain stretching between branch points is responsible for strain hardening. The figure demonstrated that the strain to show the upward departure was around 0.5, which was independent of the strain rates. As mentioned in the introduction, such rheological properties are essential for various processing operations.

## 3. Materials and Methods

A polyamide resin with low crystallinity (PA) was used. Its chemical composition was as follows: PA6 = 55 mol%, PA66 = 13 mol%, and PA610 = 32 mol%. Its number and weight average molecular weights were characterized by size exclusion chromatography using poly(methyl methacrylate) as the standard, and were 1.14 × 10^4^ Da and 2.46 × 10^4^ Da, respectively. An alternative *α*-olefin–maleic anhydride copolymer (O-MAH; DIACARNA 30M; Mitsubishi Chemical Corp., Tokyo, Japan) was employed and is depicted in Figure 1 [29].

Before melt-mixing, the PA and O-MAH were dried under vacuum for 4 h at 65 °C. They were then melt-mixed using an internal batch mixer (Labo Plastomill; Toyo Seiki Seisaku-sho Ltd., Tokyo, Japan) at 140 °C for 3 min. The blade rotation speed was 30 rpm. The PA/O-MAH weight fractions were 100/0, 30/1, and 90/10. The obtained blends were compressed into flat films using a compression-molding machine at 140 °C for 1 min, and subsequently cooled at 30 °C for 3 min.

The reaction of O-MAH was evaluated by attenuated total reflectance Fourier-transform infrared (ATR-IR) spectroscopy (Spectrum100; PerkinElmer Co., Ltd., Waltham, MA, USA) from 400 to 4000 cm^−1^. KRS-5, i.e., thallium bromoiodide, was used as an ATR crystal. The accumulation count and the resolution were 16 times and 4 cm^−1^, respectively.

The light transmittance of each film (0.3 mm thick) was measured at 25 °C using a UV-Vis spectrometer (Lambda 25; PerkinElmer Co., Ltd., Waltham, MA, USA) within the wavelength range of 200 to 800 nm.

Thermal analysis was conducted using a differential scanning calorimeter (DSC) (DSC8500; PerkinElmer Co., Ltd., Waltham, MA, USA) under a nitrogen atmosphere. Each sample was placed in an aluminum pan and heated from 25 to 140 °C at a rate of 10 °C min^−1^, then cooled at 10 °C min^−1^. Each sample weighed approximately 5 mg.

The temperature dependencies of the tensile storage moduli *E*′, loss modulus *E*″, and loss tangent *tan δ* were evaluated from −80 to 140 °C using a dynamic mechanical analyzer (Rheogel-E4000; UBM Co., Ltd., Muko, Japan). The measurements were performed at a frequency of 10 Hz within the linear viscoelastic region. The heating rate was 2 °C min^−1^. Rectangular samples cut from the compression-molded films (5 mm wide, 7 mm long, and 1 mm thick) were employed for the measurements.

The melt density of the PA was measured under various pressures using a pressure–volume–temperature (PVT) measuring machine (PVT Test system; Toyo Seiki Seisaku-sho Ltd., Tokyo, Japan) at 230 °C.

The angular frequency dependencies of the shear storage and loss moduli (*G*′ and *G*″, respectively) were evaluated at various temperatures using a rotational rheometer (AR2000ex; TA Instruments, Inc., New Castle, DE, USA) with a cone-and-plate geometry. The diameter of the cone was 25 mm and the cone angle was 4°. The shear stress and primary normal stress difference under steady shear were also measured using this machine at 130 °C. The transient uniaxial elongational viscosity was evaluated using the rheometer equipped with a universal testing platform (SEG2-G; Xpansion Instruments LLC, Tallmadge, OH, USA) at 130 °C. Rectangular specimens (10 mm wide, 15 mm long, and 1 mm thick) were used for the measurements.

Capillary extrusion was performed using a pressure-driven capillary rheometer (140-SAS-2002; Yasuda-Seiki Seisaku-sho Ltd., Nishinomiya, Japan) at 130 °C to evaluate the steady-state shear viscosity and the extrusion processability. A circular die with a length of 10 mm and a diameter of 1 mm was employed. The entrance angle of the die was 180°.

## 4. Conclusions

Polyamide resins generally have a narrow molecular weight distribution and no long-chain branches. These properties cause difficulties in various processing operations due to poor melt elasticity, including an absence of strain hardening in the transient elongational viscosity. In the present study, the rheological properties were evaluated over a wide temperature range using a polyamide resin with low crystallinity. The basic viscoelastic properties of the pure PA, such as the *G_N_*^0^ and *M*_e_, were also determined. The present study verified that the addition of O-MAH successfully modified the rheological properties of the resin through chemical reactions between the amide groups and the maleic anhydride groups. Because O-MAH is not a block copolymer, macroscopic phase separation, which causes light scattering, was not detected. The addition of O-MAH provided a PA with long-chain branches. Consequently, the melt elasticity, i.e., the primary normal stress difference and strain hardening in the transient elongational viscosity, was greatly enhanced. Although the shear viscosity increased to some degree, the extrusion performance was good and there were no flow instabilities. O-MAH is a solid at room temperature and is easy to handle. Therefore, the technique described herein can be applied to various processing operations without any difficulty.

## Data Availability

Data will be available upon request.
